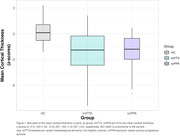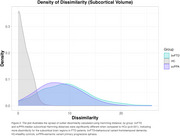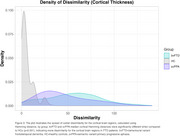# Individual‐specific brain signatures in sporadic frontotemporal dementia

**DOI:** 10.1002/alz70856_099273

**Published:** 2025-12-24

**Authors:** Kirsten W. R. Schroder, Enrico Premi, Valeria Bracca, Anthi Papouli, Rosa Manenti, Giuliano Binetti, Roberto Gasparotti, Andre F. Marquand, James H. Cole, Barbara Borroni, Martina Bocchetta

**Affiliations:** ^1^ Dementia Research Centre, UCL Queen Square Institute of Neurology, University College London, London, United Kingdom; ^2^ Stroke Unit, ASST Spedali Civili Brescia, Brescia, Italy; ^3^ Department of Clinical and Experimental Sciences, University of Brescia, Brescia, Italy; ^4^ UCL Hawkes Institute, Department of Computer Science, University College London, London, United Kingdom; ^5^ Neuropsychology Unit, IRCCS Istituto Centro San Giovanni di Dio Fatebenefratelli, Brescia, Italy; ^6^ MAC‐Memory Clinic and Molecular Markers Laboratory, IRCCS Istituto Centro San Giovanni di Dio Fatebenefratelli, Brescia, Italy; ^7^ Neuroradiology Unit, University of Brescia, Brescia, Italy; ^8^ Donders Institute for Brain, Cognition, and Behaviour, Radboud University, Nijmegen, Netherlands; ^9^ UCL Hawkes Institute, University College London, London, United Kingdom; ^10^ UCL Queen Square Institute of Neurology, University College London, London, United Kingdom; ^11^ Molecular Markers Laboratory, IRCCS Istituto Centro San Giovanni di Dio Fatebenefratelli, Brescia, Italy; ^12^ Dementia Research Centre, UCL Queen Square Institute of Neurology, University College London, London, London, United Kingdom

## Abstract

**Background:**

Frontotemporal dementia (FTD) is a heterogeneous neurodegenerative disease. Its variability in pathology and symptoms makes individual prognosis and diagnosis difficult. Most neuroimaging research assumes homogeneity within cohorts, but novel neuroanatomical modelling now allows for the estimation of individual deviations in brain regions from the norm. For the first time, this study aims to quantify patterns of neuroanatomical dissimilarity in patients with sporadic FTD.

**Method:**

T1‐weighted brain MR images of 355 participants (healthy controls ‐ HC: *n* = 116, 78 females, mean age=51.16 years; patients with behavioural variant FTD – bvFTD: *n* = 197, 78 females, mean age=66.43 years; patients with semantic variant primary progressive aphasia – svPPA: *n* = 42, 23 females, mean age=63.85 years) were processed using FreeSurfer v.6.0.0, and the output was visually inspected. Cortical thickness and subcortical volumes across 187 regions were extracted and used as input for a normative model, with a reference dataset of ∼58,000 healthy individuals. 70% of HCs were used to adapt the Bayesian linear regression algorithm, accounting for age, sex, and site. The remaining 30% of HCs and all patients were used as testing data. Regions with a z‐score <‐1.96 were classified as outliers. Normative modelling steps were completed using PCNtoolkit (v.0.31), and statistical analyses were performed using R Studio v.4.4.2.

**Result:**

Mean (SD) cortical thickness z‐scores were ‐2.01(2.18), ‐2.04(1.94), 0.16(1.22) for bvFTD, svPPA and HCs (Figure 1). bvFTD and svPPA groups exhibited significantly more dissimilarity in cortical and subcortical regions compared to HCs (one‐way ANOVAs F(2,271)=100.62; 123.81, respectively; Tukey post hoc tests both *p* <0.001) (Figure 2 and 3). Regions with the highest proportion of outliers were the right inferior temporal gyrus for bvFTD (54%), and the left anterior, inferior and superior‐lateral temporal gyri for svPPA (>83%). The left amygdala and hippocampus were the subcortical structures with the most outliers (45% and 47% in bvFTD; 64% and 67% in svPPA, respectively).

**Conclusion:**

These results demonstrate that bvFTD and svPPA patients exhibit greater heterogeneity in subcortical and cortical regions compared to HCs, particularly in the right inferior temporal gyrus and left lateral temporal gyrus, respectively. Analysis of other FTD variants and correlations with cognitive and clinical data are ongoing.